# A new approach for investigating the relative contribution of basal glucose and postprandial glucose to HbA1_C_

**DOI:** 10.1038/s41387-021-00156-1

**Published:** 2021-06-04

**Authors:** Jing Ma, Hua He, Xiaojie Yang, Dawei Chen, Cuixia Tan, Li Zhong, Qiling Du, Xiaohua Wu, Yunyi Gao, Guanjian Liu, Chun Wang, Xingwu Ran

**Affiliations:** 1 Innovation Center for Wound Repair, Diabetic Foot Care Center, Department of Endocrinology and Metabolism, West China Hospital, Sichuan University, Chengdu, China; 2 Department of Endocrinology and Metabolism, The First People’s Hospital of Longquanyi District, Chengdu, China; 3 Wannian Community health center in Chenghua district, Chengdu, China; 4 Shudu Community health center in Xindu district, Chengdu, China; 5 Chinese Cochrane Centre, Chinese EBM Centre, West China Hospital, Sichuan University, Chengdu, China

**Keywords:** Diabetes, Diabetes

## Abstract

To develop an accurate method for evaluating the relative contributions of basal glucose (BG) and postprandial glucose (PPG) to glycated haemoglobin (HbA1c) in subjects with hyperglycaemia using a Continuous Glucose Monitoring System (CGMS®). The subjects were divided into the normal glucose tolerance (NGT), impaired glucose tolerance (IGT), newly-diagnosed type 2 diabetes (NDDM), and drug-treated type 2 diabetes (T2DM) groups. We evaluated the relative contributions of BG and PPG to HbA1c in patients with hyperglycaemia according to three different baseline values. Subjects (*n* = 490) were grouped as follows: 92 NGT, 36 IGT, 131 NDDM, and 231 T2DM. The relative contributions of PPG to HbA1c were calculated using baseline values of 6.1 mmol/L, 5.6 mmol/L, and the 24-h glucose curve of the NGT group. The relative contribution of PPG to HbA1c decreased progressively from the IGT group to the T2DM group. Compared with the 24-h glucose curve as the baseline, the relative contribution of PPG was overestimated in 9.04% and 1.76% of the subjects when 6.1 mmol/L and 5.6 mmol/L were used as baselines, respectively (*P* < 0.01), in T2DM patients. The 24-h glucose curve of NGT is more suitable for studying the relative contributions of BG and PPG to HbA1c and it is more precise, as it considers physiological fluctuations in NGT after meals. However, 5.6 mmol/L can be used when the 24-h glucose curve for NGT is unavailable; using 6.1 mmol/L as a baseline value may overestimate the contribution to the HbA1c. There is no unified standard for assessing the contributions of basal glucose (BG) and postprandial glucose (PPG) to HbA1c. The 24-h glucose curve of NGT is more suitable for studying the relative contributions of BG and PPG to HbA1c, as it considers physiological fluctuations in NGT after meals. However, 5.6 mmol/L can be used when the 24-h glucose curve for NGT is unavailable; using 6.1 mmol/L as a baseline value may overestimate the contribution to the HbA1c.

## Introduction

Glycaemic control is a cornerstone in reducing the morbidity and mortality of diabetes. Previous studies have focused on haemoglobin A1c (HbA1c) and fasting plasma glucose (FPG)/basal glucose (BG) values to determine the level of glycaemic control. Studies have demonstrated that only comprehensive control of HbA1c, BG, and postprandial glucose (PPG) can prevent the occurrence and progression of vascular complications^1^. The United Kingdom Prospective Diabetes Study showed that FPG and HbA1c are the main risk factors for cardiovascular complications^2^, and reduced FPG levels are associated with reduced cardiovascular mortality in diabetes^3^. However, other studies showed that PPG is associated with cardiovascular events and all-cause mortality^4,5^.

In 2003, Monnier et al.^6^ first proposed that FPG and PPG contribute to HbA1c in patients with type 2 diabetes mellitus (T2DM) being administered antidiabetic treatment except for insulin and acarbose. Since the first milestone article was published, an increasing number of researchers had addressed these relationships. However, the studies were conducted using various methods and yielded different results. Riddle et al.^7^ analysed data from six randomised controlled trials and found that the relative contribution of BG played a major role (76–80%) when the HbA1c was >7.0%. However, the A1_C_-derived average glucose study^8^ showed that BG was not a clear indicator of general glycaemia. Table 1 summarizes 17 relevant studies^6,7,9–25^ published up to Jan 1, 2021. It shows the complex results on the relative contribution of PPG to HbA1c in diverse subjects, treatment regimens, and baseline criterias. Eleven articles used 5.5/5.6 mmol/L as baseline value. The value of 5.6 mmol/L (100 mg/dL) was chosen to align with the recommendation of the American Diabetes Association for the upper limit of normal fasting glucose levels^26^. Four articles utilized 6.1 mmol/L(110 mg/dL) as a baseline value, which has been described as the upper limit of normal fasting glucose levels according to the World Health Organization^27^. Two articles only discussed the correlation between PPG and HbA1c, without the specific contribution of each factor^10,12^. Moreover, one study firstly used the 24-h glucose profiles of the NGT curve as a baseline value, firstly pointed and discussed the influence of different baseline values, and found that the relative contribution of PPG may overestimated by approximately 10–20% when 6.1 mmol/L was used as a baseline, compared with the 24-h glucose profiles of the NGT curve^17^. Bergenstal^28^ stated that CGM has transformed glucose control and can be used to identify glucose excursions in patients with diabetes. The recent development of some new therapies specifically aimed at reducing BG or PPG has further increased the interest for studying the complex relationship between the contributions of BG and PPG, yet have yielded conflicting results^20,21,23,25^. Recently, the results of a randomised crossover trial conducted by CGMS pointed that the conflicting results may be related to differences in the study population, methodologies, etc^29^. Therefore, this study was conducted to develop a more accurate method for evaluating the relative contributions of BG and PPG to HbA1c by using a CGM.

**Table 1:** Recent studies on the relationship between BG, PPG and HbA1c.

Study	*N*	Type of patients			Threshold (mmol/L)	Methods		HbA1c(%)	The relative contributions of PPG (%)
Monnier et al.^6,14^ (2003)	290	(a) Type 2 diabetes on diet control, SU or MTF	(b) Non-insulin & non-AGI treated	(c) Caucasians	≥6.1	(a) Four-point	(b) Two standardized meals	6.3–11.4	From the lowest (69.7%) to the highest quintile of HbA1c (30.5%).
Riddle et al.^7^ (2011)	1,699 (6 trials)	(a) Type 2 diabetes on MTF/SU or both	(b) Add on basal or premix insulin	(c) 94.6% Caucasians	≥5.6	(a) Seven-point SMBG	ND	8.7(7.6–10.0)	The relative contributions of BG played a major role before intensive treatment.
Woerle et al.^9^ (2007)	164	(a)Type 2 diabetes on diet control, MTF,SU,NPH, and others	(b) ND	(c) Caucasians	≥5.6	(a) Seven-point SMBG	(b) Three carbohydrate containing meals	8.7 ± 0.1	The relative contributions of PPG accounted for nearly 90% when HbA1c < 6.2% but only about 40% when HbA1c > 8.9%.
Shimizu et al.^10^ (2008)	57	(a) 15 Type 1 diabetes, 42 Type 2 diabetes	(b) Premix or basal bolus Insulin treated	(c) Japanese	ND	(a) Six-point SMBG	ND	ND	ND (PPH had better correlation with HbA1c)
Peter et al.^11^ (2008)	52	(a) Type 2 diabetes on gliclazide, MET or both	(b) Insulin or acarbose treated	(c) Caucasians	≥5.6	Frequently sampled blood tests	(b) standard 500-kcal mixed meal (58% carbohydrate, 22% fat and 20% protein)	7.7 ± 1.0	The relative contributions of PPG accounted for nearly 85.8% when HbA1c < 7.3% but only about 48.6% when HbA1c > 8.0%.
Kikuchi et al.^12^ (2010)	66	(a) Type 2 diabetes on diet control, MTF/ PIO/SU or basal insulin	(b) Not on prandial or premix insulin & non-AGI treated	(c) Japanese	ND	(a) Six-point SMBG	(b) Three standardized meals	5.7–12.5	ND (PPG strongly correlated at HbA1c < 8.0% and vice versa for BG)
Wang et al.^13^ (2011)	121	(a) Type 2 diabetes on MTF and SU/AGI	(b) Non-insulin treated	(c) Chinese	≥5.6	(a) 3-day CGM	(b) Three mainrecorded meals	5.7–12.7	from 71% to 44% as the increased of HbA1c, up to 70% when HbA1c < 7.1%, about 50% each when HbA1c > 7.6%.
Peter et al.^15^ (2013)	52	(a) Type 2 diabetes on SU/MTF, or both	(b) Non-insulin treated	(c) Caucasians	≥5.6	(a) Multiple venous blood for 4 h after each meal	(b) Three standardized meals	5.9–9.6	Greater BG across the HbA1c range after adjusted for nocturnal hypoglycemia. Greater PPG at HbA1c < 7.0% while greater BG at HbA1c ≥ 7.0%.
Fysekidis et al.^16^ (2013)	70	(a) Obesity, BMI > 25 kg/m^2^ (35.2 ± 6.8 kg/m^2^)	(b) Without known glycemic disorder	(c) Caucasians	≥5.5	3-day CGM	Three standardized meals	5.9 (5.1–7.4)	The relative contributions of PPG accounted for nearly 81.2% when HbA1c < 5.3% but only about 57% when HbA1c > 6.6% in overweight/obese patients.
Kang et al.^17^ (2015)	59	(a) Newly diagnosed Type 2 diabetes	(b) Drug-negative	(c) Chinese, China	≥6.1 and NGT curve	(a) 3-day CGM	(b) Three standardized meals	8.0(5.6–13.2)	77.2% (HbA1c ≤ 7.0%), 55.4%(7%<HbA1c ≤ 9.0%), 22.8%(HbA1c > 9.0%). Overestimated for 10-20% choosing 6.1 mmol/L as baseline compared with NGT curve.
Wang et al.^18^ (2017)	228	(a) Type 2 diabetes on all treatment regimes	(b) 37.7% with OAD and 62.3% with insulin	(c) Chinese, China	≥5.6	(a) 3-day CGM	ND	8.4 ± 2.1	The relative contributions of PPG accounted for nearly 62.4% when HbA1c < 6.5% but only about 35.8% when HbA1c > 8.0%.
Lim et al.^19^ (2017)	100	(a) Type 2 diabetes on all treatment regimes	(b) 58% with insulin	(c) Multi-ethnic (Malays, Indians, Chinese)	≥5.6	(a) 6-day CGM	(b) All recorded meals	6.0–14.0	From 49% (HbA1c < 7.0%) to 39% (HbA1c ≥ 10.0%), greater PPG contribution when HbA1c was <8%. BG predominated when HbA1c was ≥9and ≥ 10% in oral antidiabetic drug- and insulin-treated patients.
Li et al.^20,22^ (2018)	81	(a) T2DM treated with insulin	(b) LM25 and LM50	(c)Chinese, China	≥6.1	(a) 4-point blood glucose profile at least one day	(b) high-carbohydrate test meals on day 1 and high-fat test meals on day 2 and their habitual diets on day 3	7.8 ± 1.4	PPG played a major role of patients with HbA1c < 8.5%. PPG’s contribution rate decreased with increasing HbA1c (from 65.5% to 39.2%).
Reznik et al.^21^ (2018)	259	(a) T2DM treated by insulin	(b) Non-oral drug treated	(c) Multicenter study (Canada, Europe, United States, Africa)	≥5.6	(a) OPT2mise trial	(b) maintained efforts at lifestyle and dietary management, but carbohydrate counting was not required	8.9 ± 0.8	PPG accounts for only 20% to 30% of overall hyperglycaemia, regardless of the baseline HbA1c level who fail to respond to an intensified MDI regimen.
Umpierrez et al.^23^ (2019)	673	(a) Type 2 diabetes on dulaglutide	(b) ND	(c) Caucasians	≥5.6	(a) Seven-point SMBG	ND	8.1 ± 0.94	The relative contributions of PPG accounted for nearly 52% when HbA1c < 7.0% but only about 23% when HbA1c ≥ 9%.
Yan et al.^24^ (2019)	305	(a)Newly diagnosed type 2 diabetes or IGT/IFG	(b) Drug-native	(c) Chinese	≥6.1	(a) 3-day CGM	(b) Personalized instructions and meals of a total daily caloric intake of 25 kcal/kg/day	9.3 ± 1.9	Regression analyses indicate that the contributions of FG and PG were equal (both 50%) when the level of HbA1c was 8.5%. FG contributed signifificantly more than PG in the higher groups of HbA1c (9.6-10.9% and 11.0-14.6%).
Moon et al.^25^ (2020)	194	(a)Type 2 diabetes on diet control, MTF,SU or DPP4i	(b) Non-insulin & non-AGI treated	(c)Korean	≥5.6	(a) Seven-point SMBG	ND	7.0 ± 0.9	The relative contribution of PPG decreased (55.3%±5.5%, 42.0%±4.4%, 33.5%±2.8%) with the elevated HbA1c(≤6.6%,6.7%-7.1%,≥7.2%). HbA1c, waist circumference, and triglyceride had a significant association with AUC_FHG_. Only HbA1c and age was associated with AUC_PPG_.

## Materials and methods

### Population and subject selection

Subjects with a normal glucose tolerance (NGT), impaired glucose tolerance (IGT), newly diagnosed type 2 diabetes (NDDM), and drug-treated type 2 diabetes (T2DM) from multiple communities including the Chenghua, Xindu, and Longquanyi districts in Chengdu city and West China Hospital of Sichuan University were consecutively enrolled in this study. The study was approved by the Biomedical Research Ethics Committee of West China Hospital of Sichuan University. All participants provided written informed consent.

All participants were 18–75 years of age. Participants in the NGT group were in good health without obesity, hyperlipidaemia, diabetes, or hypertension. Normal blood pressure was defined as 90–139/60–89 mmHg, and normal lipid profiles included triglyceride <2.22 mmol/L, total cholesterol <6.22 mmol/L. The body mass indices were 18.5–24.9 kg/m^2^. Drug-treated patients met the diagnostic criteria for T2DM according to the World Health Organization^27^ standard over 6 months and were treated with fixed oral anti-diabetic drugs for at least 3 months before the study. Participants who had impaired liver (aspartate aminotransferase or alanine aminotransferase levels 2-fold the upper the limit of the normal range) or renal (serum creatinine 1.2-fold the upper normal range or estimated glomerular filtration rate <30 mL/min) function or had other comorbidities or conditions that may lead to severe blood glucose fluctuation, such as malignant tumours, human immunodeficiency virus infection, acute infections, trauma, or surgery were excluded from the study. Insulin, DPP-4 inhibitors, GLP-1 receptor agonists, and acarbose treatments were also excluded to avoid any bias in the interpretation of the contribution of PPG increments to overall hyperglycaemia, as they exert specific effects on PPG excursions.

### Outcome measures

Medical history data and anthropometric data (blood pressure, height, weight, etc.) were recorded. An oral glucose tolerance test(OGTT) was performed for each participant. The plasma glucose concentration was measured by the glucose oxidase method. HbA1c was measured by high-performance liquid chromatography (Bio-Rad-10, Hercules, CA, USA). Biochemical parameters, including triglyceride, total cholesterol, high-density lipoprotein-cholesterol, low-density lipoprotein-cholesterol, liver function, and renal function were evaluated with an automatic biochemical analyser (D/P/ISE, Roche, Basel, Switzerland).

### Formulation of mixed-meals

All subjects received nutritional assessments and dietary instructions from the nutritionist and endocrinologist of our hospital based on the China Medical Nutritional Therapy Guideline for Diabetes (2013)^30^. Adjusted the individualized energy standard mainly according to the subject’s BMI, age, activity level, and then individualized dietary recipes plan was formulated. The ratio of carbohydrate, proteins, and fats were 45–60%, 15–20%, and 25–35%. For overweight or obese subjects, the ratio of fat was controlled within 30% (Supplementary Table 1). Subjects were instructed to have breakfast, lunch, and dinner at 7:00–8:00AM, 11:00–12:00 AM, and 5:00–6:00 PM, respectively. Subjects were not permitted to eat between meals and were required to record the mealtime, amount, cooking style and species of each meal carefully. In this study, we adopted a relatively fixed-time, fixed-ratio diet to avoid bias caused by differences in diet. All participants refrained from consuming alcohol, strong tea, and coffee during the monitoring period^30,31^.

### Installation of CGMS

CGMS® Gold (Medtronic MiniMed, Northridge, CA, USA) was administered for CGM in this study. The glucose sensor was inserted into the subcutaneous tissue of the abdomen of all participants at 08:00–09:00 in the morning to monitor the glucose levels in the interstitial fluid for at least 72 h. Finger stick blood glucose levels were checked to calibrate and four calibrations per day (before meals and at bedtime) were conducted with an Accu-Chek Integra Blood Glucose Meter (Roche) during the CGM period. The subjects returned to our hospital to download the data from CGMS after 3 days. The data analysed using the Solution Software v3.0c.

### Calculation method and three baseline criteria

**Fig. 1:**
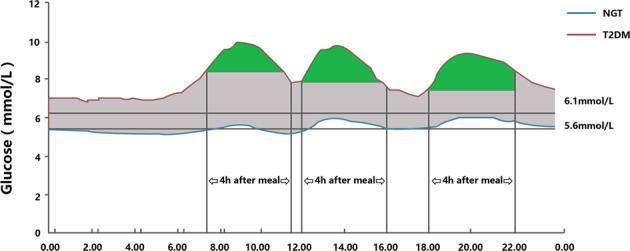
Calculationused for the AUC under condition A. The green part represents AUC-_PPG_. The gray partrepresents AUC-BG (AUC-T – AUC-PPG).

**Fig. 2:**
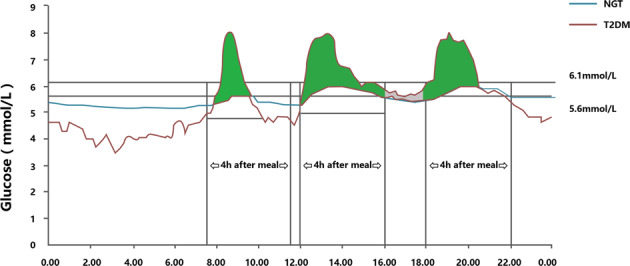
Calculation used for the AUC under condition B. If the glucose profile was under the baseline, ascondition B. AUC-T was only calculated using glucose values above the baseline criteria, and the area below the baseline point was not included in the calculation of overall hyperglycaemia. The green part represents AUC-PPG. The gray part represents AUC-BG (AUC-T– AUC-PPG).

The overall additional hyperglycemia, which can be further divided into two subcomponents: the basal glucose (BG) and postprandial glucose (PPG). One question to be raised is to define the word “Basal glucose”, with the word “basal” being probably more appropriate than “fasting”. As soon as these two components of hyperglycemia were identified, diabetologists have paid increasing attention to their respective contributions to the overall hyperglycemia in hyperglycemia subjects. BG is the component that remains after the PPG has been subtracted from the overall hyperglycemia. Three baseline criterias, which were 6.1 mmol/L, the upper limit of fasting blood glucose in normal subjects according to the World Health Organization standard^27^ in method A; 5.6 mmol/L, the upper limit of fasting blood glucose in normal subjects according to the American Diabetes Association standard^26^ in method B and 24-h glucose profiles of the NGT curve as baseline in method C, respectively, were performed to evaluate the contribution of PPG to HbA1c. The area under the curve and above the baseline criteria represented excessive hyperglycaemia, named as AUC_-T_. This value was calculated as AUC_-T_ = AUC_T2DM_ - AUC_baseline_ when the glucose profile of subjects was always higher than that of the baseline value, as condition A (Fig. 1). However, if the glucose profile was under the baseline, as condition B (Fig. 2), AUC_-T_ was only calculated using glucose values above the baseline criteria, and the area below the baseline point was not included in the calculation of overall hyperglycaemia. AUC_-PPG_ reflects the 4-h glucose incremental area above the pre-prandial glucose values assessed just before starting breakfast, lunch, and dinner, and AUC_-PPG-Total_ = AUC_-PPG-b_ + AUC_-PPG-l_ + AUC_-PPG-d_. The contribution of PPG to overall hyperglycaemia was calculated as AUC_-PPG-Total_/AUC_-T_×100%. The contribution of BG to overall hyperglycaemia was calculated as (AUC_-T_ − AUC_-PPG-Total_)/AUC_-T_ × 100%.

### Statistical analysis

Statistical analyses were performed using SPSS 21.0 software (SPSS, Inc., Chicago, IL, USA). Data were expressed as the means ± SDs or median with the quartile range. One-way analysis of variance was used among the studied groups to test differences in normally distributed data. The contributions of PPG under the three different baseline criteria were analysed using the Wilcoxon signed rank test between groups. All tests were 2-sided, and *p*-values below 0.05 were considered as statistically significant.

## Results

### Clinical and laboratory data

A total of 490 subjects were enrolled in this study, including 92 NGT, 36 IGT, 131 NDDM, and 231 T2DM subjects. Table 2 shows the clinical and laboratory data of all subjects. The average age was 53.03 ± 12.00 years (21–77 years) and average HbA1c value was 6.96 ± 1.26% (4.5–12.7%). The blood lipid parameters were in the normal reference value range, however, from the IGT group to the NDDM group, age, body mass index, triglyceride, total cholesterol, low-density lipoprotein-cholesterol were also gradually increased (*p* < 0.05). The NDDM group showed higher levels of systolic blood pressure, diastolic blood pressure, HbA1c, triglyceride, total cholesterol, low-density lipoprotein-cholesterol parameters, and a lower level of high-density lipoprotein-cholesterol compared to those in the T2DM group after drug treatment.

**Table 2:** The clinical and laboratory data of all subjects.

	NGT(*n* = 92) 95% CI	IGT(*n* = 36) 95% CI	NDDM(*n* = 131) 95% CI	T2DM(231) 95% CI	Total(*n* = 490) 95% CI	*p*
Age(yr)	43.28 ± 15.44	51.06 ± 12.14	54.54 ± 9.39	56.36 ± 9.40	53.03 ± 12.00	<0.001
	(40.09, 46.48)	(46.95, 55.16)	(52.91, 56.16)	(55.14, 57.58)	(51.96, 54.09)	
Gender(M/F)	41/51	13/23	76/55	104/127	234/256	**0.036**
HbA1c(%)	5.7 ± 0.41	6.00 ± 0.37	7.56 ± 0.84	7.26 ± 1.35	6.96 ± 1.26	<0.001
	(5.61, 5.79)	(5.87, 6.12)	(7.42, 7.71)	(7.09, 7.44)	(6.84, 7.07)	
BMI(kg/m2)	22.03 ± 2.20	23.45 ± 2.28	24.82 ± 2.90	25.54 ± 3.28	24.54 ± 3.22	<0.001
	(21.57, 22.48)	(22.68, 24.22)	(24.32, 25.33)	(25.11, 25.96)	(24.25, 24.82)	
SBP(mmHg)	115.18 ± 17.11	120.11 ± 14.91	127.88 ± 14.88	127.82 ± 16.70	124.90 ± 16.92	<0.001
	(111.64, 118.73)	(115.07, 125.16)	(125.31, 130.45)	(125.66, 129.99)	(123.40, 126.40)	
DBP(mmHg)	76.25 ± 9.67	78.44 ± 8.13	79.49 ± 9.22	77.52 ± 9.98	77.87 ± 9.64	**0.08**
	(74.25, 78.25)	(75.69, 81.20)	(77.89, 81.08)	(76.22, 78.81)	(77.02, 78.73)	
TG(mmol/L)	1.03 ± 0.43	1.18 ± 0.49	1.86 ± 1.20	1.78 ± 1.28	1.61 ± 1.14	<0.001
	(0.95, 1.12)	(1.01, 1.35)	(1.66, 2.07)	(1.61, 1.94)	(1.52, 1.72)	
TC(mmol/L)	4.58 ± 0.85	4.77 ± 0.70	4.88 ± 0.86	4.54 ± 0.89	4.66 ± 0.87	**0.003**
	(4.40, 4.76)	(4.53, 5.01)	(4.73, 5.03)	(4.42, 4.66)	(4.58, 4.73)	
LDL-C(mmol/L)	2.72 ± 0.66	2.82 ± 0.71	2.93 ± 0.90	2.51 ± 0.79	2.69 ± 0.81	<0.001
	(2.58, 2.85)	(2.58, 3.06)	(2.77, 3.09)	(2.41, 2.62)	(2.61, 2.76)	
HDL-C(mmol/L)	1.68 ± 0.47	1.57 ± 0.34	1.24 ± 0.34	1.38 ± 0.41	1.41 ± 0.43	<0.001
	(1.59, 1.78)	(1.46, 1.69)	(1.18, 1.30)	(1.32, 1.43)	(1.37, 1.45)	

*p* ≤ 0.05 represents a statistically significant difference.

*BMI* Body mass index, *SBP* Systolic blood pressure, *DBP* Diastolic blood pressure, *TG* Triglyceride, *TC* Total cholesterol, *HDL-C* High-density lipoprotenin cholesterol, *LDL-C* Low-density lipoprotenin cholesterol.

DBP has no statistically difference between the groups. TC gradually increased from the IGT group to the NDDM group. It has statistically difference between the groups.

### Relative contribution of PPG to HbA1c calculated by three baseline criteria

**Fig. 3:**
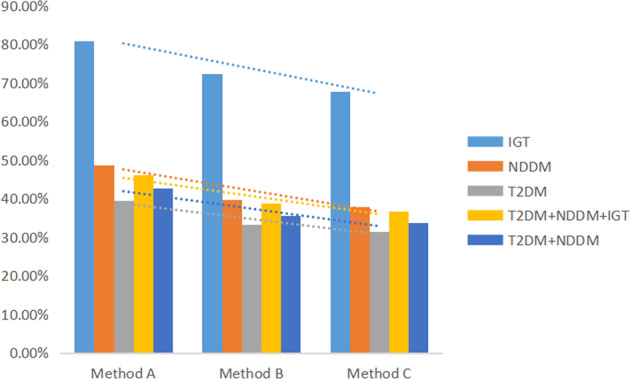
Relative contribution of PPG to overall hyperglycaemia using three methods in different groups (methods A, B, and C used 6.1 mmol/L, 5.6 mmol/L, and the 24-h glucose profiles of the NGT curve as baselines, respectively). Different colors represent different groups.

**Fig. 4:**
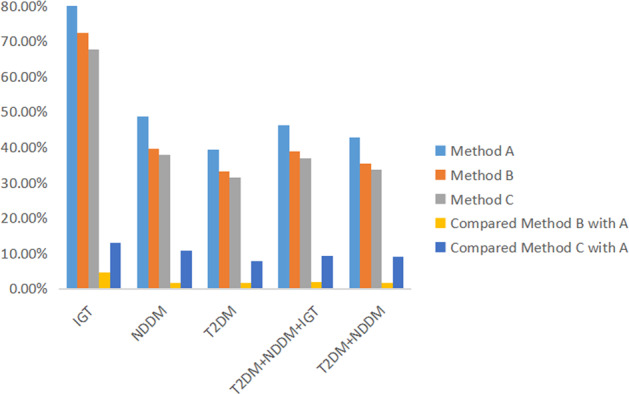
The overestimated proportion for relative contribution of PPG to overall hyperglycaemia by methods A and B, compared with baseline of the 24-h glucose profiles of the NGT in different groups.

The relative contributions of PPG according to the three baseline criteria were significant with each other among all participants (*p* < 0.01). As shown in Table 3, the relative contributions of PPG to HbA1c calculated by methods A, B, and C for IGT were 80.85%, 72.36%, and 67.68%; for NDDM were 48.76%, 39.63%, and 37.85%; for T2DM were 39.46%, 33.22%, and 31.47%; and for NDDM + T2DM were 42.82%, 35.54%, and 33.78%, respectively. Based on these results, the NGT curve reflects physiological fluctuations in normal people after each meal, which is more accurate as a baseline criterion. In all subjects with hyperglycaemia, the relative contributions of PPG decreased progressively from the IGT group to the T2DM group regardless of the baseline criteria used, whereas the relative contributions of BG increased gradually (Fig. 3). Further analysis showed that the relative contribution of PPG was overestimated by approximately 13.16% when 6.1 mmol/L was used as a baseline, compared to when the NGT curve was used as the baseline in the IGT group. It was also overestimated in NDDM and T2DM groups (*p* < 0.01) (Table 3). We also found that from the T2DM group to the IGT group, the overestimated level of the relative contribution of PPG to HbA1c also gradually increased (Fig. 4). Compared with method C, the relative contribution of PPG was overestimated by 9.04% and 1.76% when 6.1 mmol/L and 5.6 mmol/L were used as a baseline, respectively, for patients with T2DM.

**Table 3:** The comparison of the relative contribution of PPG by three baseline criterias.

	Mean	SD	P25	P50	P75	*N*	*p*1-2
							*p* 1-3
							*p* 2-3
IGT							
Contribution6.1%	80.85	21.05	61.56	87.91	100.00	36	**0.003**
Contribution5.6%	72.36	25.48	52.88	77.69	97.04	36	<0.001
Contribution NGT%	67.68	25.64	49.23	66.56	94.05	36	<0.001
NDDM							
Contribution6.1%	48.76	24.46	28.97	45.83	64.90	131	<0.001
Contribution5.6%	39.63	20.89	25.67	36.46	49.10	131	<0.001
Contribution NGT%	37.85	20.55	23.62	34.67	46.02	131	<0.001
T2DM							
Contribution6.1%	39.46	28.22	17.57	31.12	59.94	231	<0.001
Contribution5.6%	33.22	24.46	14.13	26.12	49.09	231	<0.001
Contribution NGT%	31.47	23.67	13.18	24.71	45.66	231	0.003
IGT + T2DM + NDDM							
Contribution6.1%	46.26	28.88	22.95	41.69	68.51	398	<0.001
Contribution5.6%	38.87	25.83	18.33	32.98	54.25	398	<0.001
Contribution NGT%	36.84	24.98	17.41	30.96	50.77	398	<0.001
NDDM + T2DM							
Contribution6.1%	42.82	27.25	21.42	37.45	61.24	362	<0.001
Contribution5.6%	35.54	23.41	17.88	29.37	49.10	362	<0.001
Contribution NGT%	33.78	22.77	16.64	28.27	45.94	362	<0.001

Wilcoxon Signed Ranks Test.

## Discussion

More and more studies focused on the relative contributions of BG and PPG to HbA1c, but different studies use different baseline criteria. Regardless of the previous milestone articles, or the two newly published articles^24,25^, because of the different baseline values selected, quite different research results have been obtained. Yet there is not any research to explore the influence with different baseline criteria on the results. This is the first prospective study to evaluate the relative contributions of BG and PPG to HbA1c in different subjects with hyperglycaemia (IGT, NDDM and T2DM) by using CGM and different baseline criteria in a real-world setting (Clinical Trials ID, NCT02648685). In this study, we again verified our previous findings^17^ and found that compared with using the NGT curve as a baseline, the relative contribution of PPG was overestimated by 9.04% and 1.76% when 6.1 and 5.6 mmol/L were used as a baseline, respectively, in subjects from the NDDM and T2DM groups, among all patients with T2DM. However, as the sample size increases, the proportion of this overestimation has shrunk to a certain extent.

Our previous study showed that subjects with NGT exhibited physiological glucose fluctuations after energy intake^31^, and thus, using a fixed value as a baseline to evaluate the BG and PPG contributions is not appropriate because it does not take into account physiological blood glucose fluctuations. Woerle et al.^9^ found that PPG is essential for achieving recommended HbA1c levels, and most studies have demonstrated that PPG plays an important role in overall hyperglycaemia in patients with good glycaemic control, whereas BG makes a greater contribution to the deterioration of glucose homeostasis. Based on previous studies^6,7,13–17^, we developed a method for quantifying the relative contribution of PPG and BG to hyperglycaemia. A recent meta-analysis^32^ of the correlations of BG and PPG with standard HbA1c showed a pooled correlation coefficient between PPG and HbA1c of 0.68 (*P* < 0.001, 95% CI; 0.56–0.75), which was slightly higher than that of BG (*r* = 0.61, *P* < 0.001, 95% CI; 0.48–0.72). The exact contributions of BG and PPG increments to overall hyperglycaemia remain controversial. The discrepancies between previously published data may have resulted from the interference of several co-variates. First, some studies used the multi-point glucose value or fingerstick glucose sample, whereas others used a CGM for quantification. The former test shows a loss of at least 10 h of blood glucose data and does not represent integrated glucose fluctuations throughout the day, and FPG cannot reveal the BG levels. Second, the baseline values in these reports were different. Most studies selected 6.1 mmol/L or 5.6 mmol/L as the baseline but none used the NGT curve as a baseline. Third, the contribution of PPG may have been overestimated if AUC_-T_ was calculated as AUC_T2DM_-AUC_baseline_ when the glucose value was under the baseline in some patients with T2DM, leading to more than a fraction of the area under the curve being subtracted. Thus, the area below the baseline point was not included in the calculation of overall hyperglycaemia. In addition, the Asian and Caucasian populations differ in various characteristics such as race and diet^33^. HbA1c changes slowly and reflects averages over months, whereas CGM reflects actual excursions in minutes. Brown et al. pointed CGM reviewed several clinical scenarios of glycaemic outcomes from CGM data that can be analyzed to describe glycaemic variability and its attendant risks of hyperglycaemia and hypoglycaemia, to enhance interpretation of treatment effect and improve clinical decision-making^34^. In our further research, we will explore the changes of these parameters which from CGM with different levels of HbA1c.

Recent studies of diabetes patients being administered insulin^20–22^ and Glucagon like peptide-1 receptor agonists (GLP-1 RAs)^23^ are a good complement to our research. These studies also revealed that BG plays a major role in the subgroup of patients with HbA1c levels > 8.5%, and the relative contribution of PPG decreased with increasing HbA1c (from 65.5% to 39.2%). The difference in the relative contribution of PPG between the studies performed by Li et al.^20^ and Riddle’s et al.^7^ may be that Riddle et al. used 5.6 mmol/L as a baseline, whereas Li et al. used 6.1 mmol/L as a baseline, leading to the overestimation of the relative contribution of PPG. The NGT curve considers physiological fluctuations in normal people after meals, and thus, can be used as a baseline to evaluate the relative contribution of PPG and BG to hyperglycaemia.

A new study on Asian populations conducted by Moon^25^ found consistent results with us, suggested that BG predominantly contributed to overall hyperglycemia at higher HbA1c levels. Moreover, when controlling for HbA1c and other factors, recommended TG and waist circumference showed a significant correlation with BG in Korean T2DM patients. It has been suggested in previous studies that TG and waist circumference increased fasting glucose by affecting insulin resistance^35^. So, some certain factors should be taken into account when concerning contribution of PPG and prescribing medications for T2DM patients.

This study provides a new approach for investigating the relative contribution of BG and PPG to HbA1c in T2DM by using the NGT curve as a baseline based on comparison of three different baselines. However, this study had some limitations. The survey mainly focused on differences between different baselines in different groups without discussing the differences based on different HbA1c levels. Additionally, this was a single-center study; this may have affected testing efficiency and may not be representative of all persons in China. Further, patients treated with insulin or more complex therapeutic interventions and those experiencing diabetes-related complications excluded from the study, and thus, the conclusions cannot be generalised to a larger population. Prospective research is needed to further understand the impact of various oral anti-diabetes treatment regimens on glycaemic control among patients with T2DM in China.

Our findings have important clinical implications, as the relative contribution changes with the degree of HbA1c, medications should be prescribed accordingly. T2DM is a progressive disease, and the intensity of hyperglycaemia control treatment should be increased accordingly with the development and refinement of CGM^36^. We know lifestyle intervention should be applied throughout the diabetes treatment process. When diet and exercise cannot effectively control the blood glucose levels, pharmacologic therapy should be provided timely^37^. In pre-diabetes, the relative contribution of PPG was 67.68% using the NGT curve as the baseline. That suggests we should focus on controlling the PPG excursion. We could take dietary intervention, or take the pills which control PPG, such as α-glucosidase inhibitors and so on. On the contrary, basal hyperglycemia plays a major role as the HbA1c level rises in T2DM. At this time, it is important to make basal hyperglycemia reaching target range. Then, such as basal insulin, metformin, sulfonylurea, DPP‐4 inhibitors or Glucagon like peptide-1 receptor agonists (GLP-1 RAs) should be considered with priority. When the BG decreased, the PPG levels would drop down with the tide. Furthermore, besides considering the level of HbA1c, some other factors should also be considered, such as TG, waist circumference, age, comorbidities and complications. Individualized treatment should be administrated properly and timely, ensuring patients can reach the goals of HbA1c, PPG and BG, at the same time minimize blood sugar fluctuations as soon as possible.

In conclusion, we confirmed that a progressive shift occurs in the respective contributions of BG and PPG with the increasing of glucose, and the contribution of BG is predominant in patients with worsening HbA1c. Moreover, using the NGT curve as a baseline to evaluate the contribution of BG/FG and PPG to HbA1c is accurate and precise, as it considers the physiological fluctuations in normal people after meals. If the NGT curve is not available, the standard of 5.6 mmol/L can be used, whereas a standard of 6.1 mmol/L does not appear to be given accurate results.

## Supplementary information

Supplementary Table 1

ORCID

Checklist-2020NUTD00191

Clinical Trails ID
