# Supplementary material for: A new approach for investigating the relative contribution of basal glucose and postprandial glucose to HbA1C
Source: Nutr Diabetes. 2021 Jun 4;11:14. doi: 10.1038/s41387-021-00156-1 (PMC8178390; doi:10.1038/s41387-021-00156-1)
Supplement: Supplementary file 1 — Supplementary Table 1 [file 41387_2021_156_MOESM1_ESM.docx]

Supplementary Table 1 Daily energy supply of adult diabetic patients kJ/kg(kcal/kg) and Glycemic Load (GL) of Common Foods

| Labor activity intensity | | Underweight | | Normal weight | | | Overweight | |
| --- | --- | --- | --- | --- | --- | --- | --- | --- |
| Heavy physical activity | | 188~209(45~50) | | 167(40) | | | 146(35) | |
| Moderate physical activity | | 167(40) | | 125~146(30~35) | | | 125(30) | |
| Light physical activity | | 146(35) | | 104~125(25~30) | | | 84~104(20~25) | |
| Resting state | | 104~125(25~30) | | 84~104(20~25) | | | 62~84( 15~20) | |
| Note 1: The standard weight refers to WHO 1999 calculation method: (male) standard weight = [height(cm)-100]*0.9) (kg) (Female) Standard weight = [Height (cm)-100]*0.9(kg)-2.5(kg).  Note 2: BMI≤18.5 means underweight, 18.5~24.0 means normal weight, ≥24~28 means overweight, ≥28.0 means obesity. | | | | | | | | |
| Food name | GL（per 100 g） | | Food name | | GL（per 100 g） | Food name | | GL（per 100 g） |
| Sticky rice | 17.8 | | Instant noodles | | 7.2 | Watermelon | | 9.9 |
| Buckwheat bread | 16.4 | | Vixen noodles | | 7. 1 | Banana | | 8. 1 |
| Rice | 16.2 | | Lotus root starch | | 6.9 | Pineapple | | 6. 3 |
| Pancakes | 14.7 | | Pumpkin | | 5.9 | Kiwi | | 6. 2 |
| Soda crackers | 13.7 | | Carrot | | 5.5 | Soy milk | | 4.9 |
| Steamed roll | 13.3 | | Mung Bean Noodles | | 5 | Apple | | 4.4 |
| Millet (boiled) | 13.3 | | Lotus seeds | | 5 | Orange | | 4.4 |
| Whole wheat bread | 12.1 | | Taro (steamed) | | 5 | Grape | | 4. 3 |
| Wheat noodles | 11.8 | | Yam | | 4.4 | Strawberry | | 4.3 |
| Ice cream | 11.1 | | Green beans | | 3.8 | Mango | | 3.9 |
| potato | 11.0 | | Watermelon | | 9.9 | Pear | | 3.7 |
| Burger buns | 10. 7 | | Rice noodles | | 3.2 | Peach | | 3.1 |
| Chestnut | 10.7 | | Potato vermicelli | | *2.7* | Skimmed milk | | 2.6 |
| Soy noodles | 9.8 | | Broad Beans | | 2.5 | Grapefruit | | 2.3 |
| Sushi | 9.6 | | Dried tofu | | 1.3 | Yogurt | | 2.3 |
| Fritters | 9.4 | | Onion | | 1.2 | Cherry | | 2.2 |
| Polenta | 9.4 | | Frozen tofu | | 0. 8 | Plum | | 1.9 |
| Buckwheat | 9.0 | | Peanut | | 0.4 | Whole milk | | 1.5 |
| Note: This data comes from the *China Food Composition Table 2002*. | | | | | | | | |
