# Supplementary figures and images for: A new approach for investigating the relative contribution of basal glucose and postprandial glucose to HbA1C
Source: Nutr Diabetes. 2021 Jun 4;11:14. doi: 10.1038/s41387-021-00156-1 (PMC8178390; doi:10.1038/s41387-021-00156-1)

Xingwu Ran <https://orcid.org/0000-0002-6634-1241>


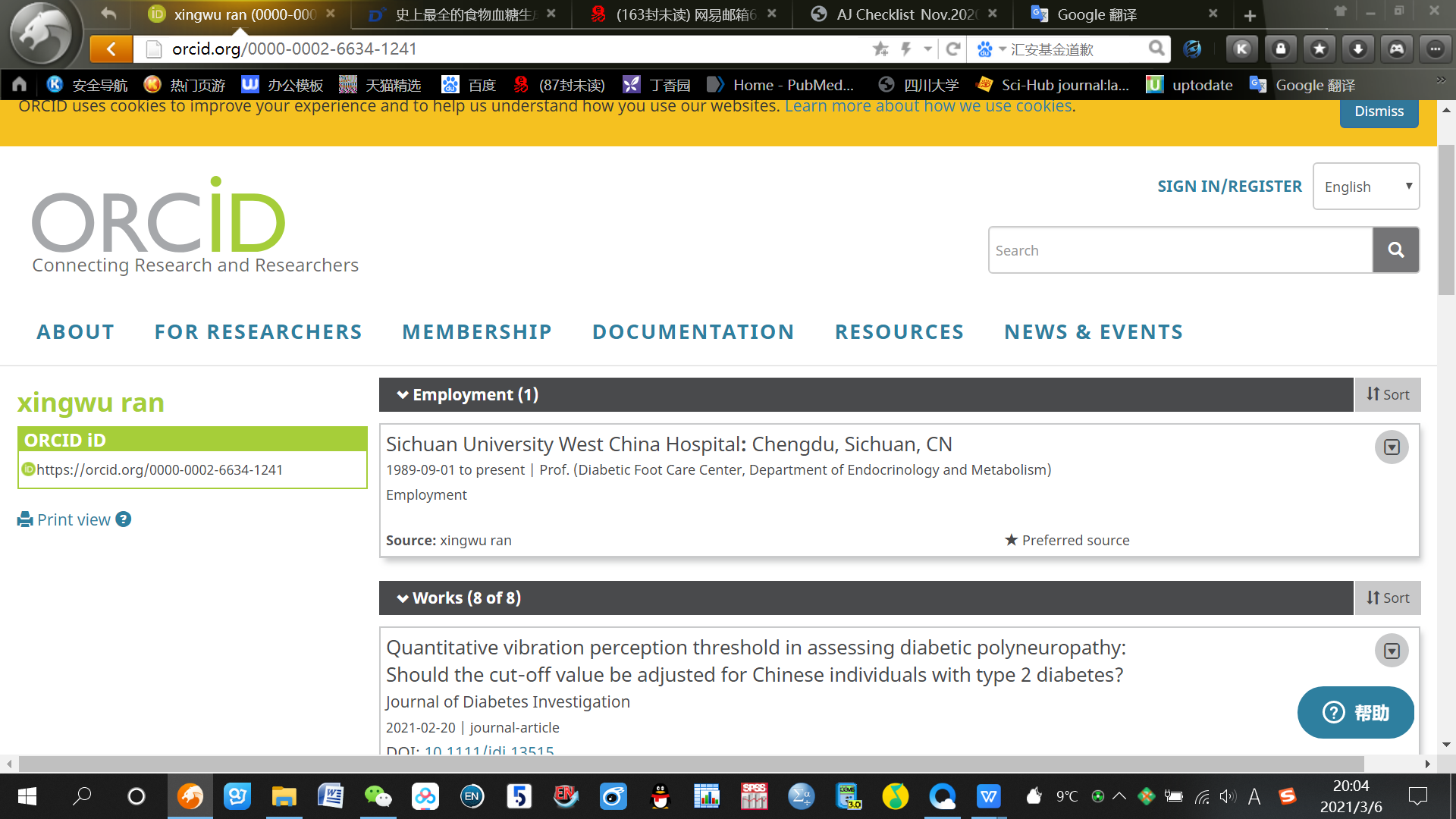


Jing Ma <https://orcid.org/0000-0001-6159-0209>


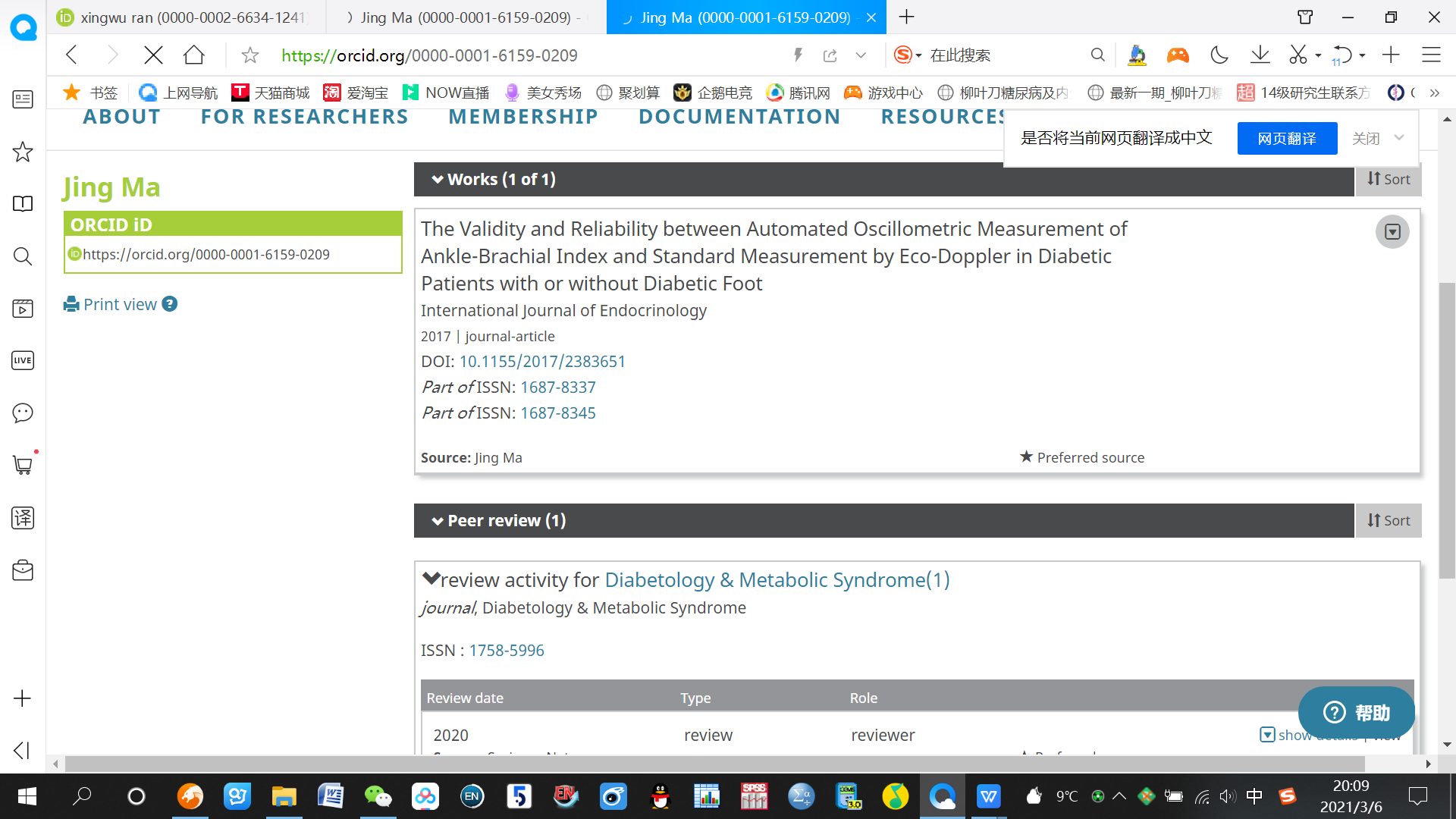

Supplement: Supplementary file 2 — ORCID [file 41387_2021_156_MOESM2_ESM.docx]
