# Supplementary material for: A new approach for investigating the relative contribution of basal glucose and postprandial glucose to HbA1C
Source: Nutr Diabetes. 2021 Jun 4;11:14. doi: 10.1038/s41387-021-00156-1 (PMC8178390; doi:10.1038/s41387-021-00156-1)
Supplement: Supplementary file 4 — Clinical Trails ID [file 41387_2021_156_MOESM4_ESM.docx]

This is the first prospective study to evaluate the relative contributions of BG and PPG to HbA1c in different subjects with hyperglycaemia (IGT, NDDM and T2DM) by using CGM and different baseline criteria in a real-world setting (Clinical Trials ID, NCT02648685).
